# Investigation of variability in image acquisition and contouring during 3D ultrasound guidance for partial breast irradiation

**DOI:** 10.1186/1748-717X-9-35

**Published:** 2014-01-27

**Authors:** Anthony Landry, Tanya Berrang, Isabelle Gagne, Carmen Popescu, Tracy Mitchell, Hazel Vey, Letricia Sand, Siew Yan Soh, Jill Wark, Ivo Olivotto, Wayne Beckham

**Affiliations:** 1 Radiation Therapy Program, Prince Edward Island Cancer Treatment Centre, Charlottetown, PE, Canada; 2 Department of Radiation Oncology, Dalhousie University, Halifax, NS, Canada; 3 Radiation Therapy Program, BC Cancer Agency – Vancouver Island Centre, Victoria, BC, Canada; 4 Division of Radiation Oncology, University of British Columbia, Vancouver, BC, Canada; 5 Departments of Physics and Astronomy, University of Victoria, Victoria, BC, Canada; 6 Prince Edward Island Cancer Treatment Centre, Queen Elizabeth Hospital, 60 Riverside Drive, PO BOX 6600, Charlottetown, PE, Canada

**Keywords:** Breast cancer, 3D ultrasound, Image guided radiotherapy

## Abstract

**Background:**

Three-dimensional ultrasound (3DUS) at simulation compared to 3DUS at treatment is an image guidance option for partial breast irradiation (PBI). This study assessed if user dependence in acquiring and contouring 3DUS (operator variability) contributed to variation in seroma shifts calculated for breast IGRT.

**Methods:**

Eligible patients met breast criteria for current randomized PBI studies. 5 Operators participated in this study. For each patient, 3 operators were involved in scan acquisitions and 5 were involved in contouring. At CT simulation (CT1), a 3DUS (US1) was performed by a single radiation therapist (RT). 7 to 14 days after CT1 a second CT (CT2) and 3 sequential 3DUS scans (US2a,b,c) were acquired by each of 3 RTs. Seroma shifts, between US1 and US2 scans were calculated by comparing geometric centers of the seromas (centroids). Operator contouring variability was determined by comparing 5 RT’s contours for a single image set. Scanning variability was assessed by comparing shifts between multiple scans acquired at the same time point (US1-US2a,b,c). Shifts in seromas contoured on CT (CT1-CT2) were compared to US data.

**Results:**

From an initial 28 patients, 15 had CT visible seromas, met PBI dosimetric constraints, had complete US data, and were analyzed. Operator variability contributed more to the overall variability in seroma localization than the variability associated with multiple scan acquisitions (95% confidence mean uncertainty of 6.2 mm vs. 1.1 mm). The mean standard deviation in seroma shift was user dependent and ranged from 1.7 to 2.9 mm. Mean seroma shifts from simulation to treatment were comparable to CT.

**Conclusions:**

Variability in shifts due to different users acquiring and contouring 3DUS for PBI guidance were comparable to CT shifts. Substantial inter-observer effect needs to be considered during clinical implementation of 3DUS IGRT.

## Background

The target for partial breast irradiation (PBI) and boost radiotherapy relies on definition of the seroma or healing surgical bed plus a margin [1,2], but the ideal method to define the target for PBI remains unclear [3]. Clinical examination is inaccurate resulting in 50-80% of the seroma receiving inadequate dose [4]. Using surgical clips to define the target has been associated with a higher recurrence rate [5], smaller volumes compared to CT-delineated volumes [6], and clips are uncommonly placed by surgeons. It has been proposed that daily cone beam CT (CBCT) of the excision cavity is optimal during external beam PBI [7], however, this is not unanimous [8,9] and daily CBCT increases integral dose.

Ultrasound (US) provides good image quality in breast tissue, is non-ionizing, and identifies the seroma in most breast cancer patients [10,11]. One distinction between 3DUS and other image-guided radiotherapy (IGRT) modalities is the user dependence of the image acquisition process that may result from scanning variability, patient respiration, probe pressure, selected image settings, and the chosen contouring strategy. In most clinical radiotherapy departments it is likely that multiple operators would be involved with the scanning and contouring tasks associated with a breast cancer patient’s US guided treatment. This study aimed to determine the extent to which operator variability in 3DUS acquisition and seroma contouring contributed to differences calculated for breast IGRT.

## Methods

### Study subjects

Eligible subjects were women undergoing CT simulation for adjuvant whole breast radiotherapy. Inclusion criteria were age ≥ 40 years, pathologically confirmed ductal carcinoma in-situ (DCIS) or invasive ductal breast cancer ≤ 3 cm diameter treated with breast conserving surgery with negative margins. Women were excluded if they had mastectomy, lobular histology, multicentric disease, bilateral breast cancer, or if their CT simulation was > 14 weeks after the last breast surgery. This study was approved by the institutional research ethics board. All subjects provided written informed consent.

### 3D ultrasound system

3DUS was performed using the Clarity (Clarity™, Elekta Soft Tissue Visualization, Montreal, Quebec) US system that consists of three components: a 10-MHz probe with a linear transducer array to collect US images; a computer system to reconstruct and display the images, and an infrared imaging system to track the location of the US probe in the CT room. The US and CT images were implicitly registered through a shared common coordinate system.

The Clarity System is equipped with two different contouring platforms: [1] ‘Workstation Mode’ , which is an off-line platform (patient not present) that allows the observer full latitude to visualize and contour the lumpectomy cavity using several manual and semi-automated contouring tools and [2] ‘Guide Mode’ , an on-line platform (patient present) which allows the observer to contour a live 3DUS acquisition using a manual approach, a 5-point semi automated contouring approach, or by overlaying the patient’s position reference volume (an imported 3DUS structure defined on previous 3DUS) on the current 3DUS scan.

A daily quality assurance program was developed and implemented to ensure the integrity of the Clarity System within the CT simulator room was accurate to within 1.0 mm.

### Image acquisition

#### Simulation (CT1 and US1)

**Figure 1:**
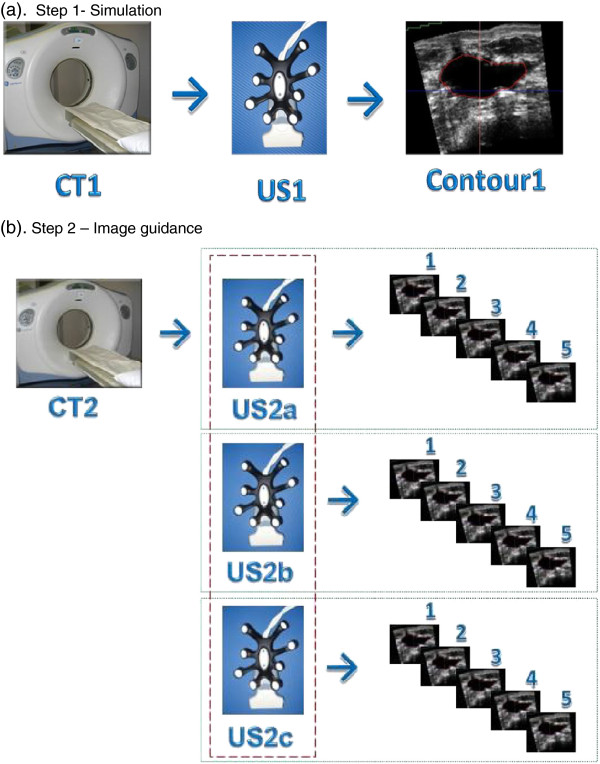
**Image acquisition and guidance process. (a)** Imaging acquisition process at simulation. A simulation CT was acquired (CT1) followed by a single 3DUS scan (US1). US1 picture shows the 3D US probe. CT1 was contoured offline by a single RO while US1 was contoured by a single RT. **(b)** Image guidance process at ‘treatment’. A second CT was acquired (CT2) followed by three subsequent 3DUS scans made by three different RTs (US2a, US2b, US2c). CT2 was contoured by a single RO and a seroma shift was determined based on implicit registration of CT coordinates. US2a, US2b, and US2c were each contoured by 5 RTs and a seroma shift was calculated by comparing each contour with the initial US1. This study design allowed for an analysis of scanning variability (dashed line) and contouring variability (dotted line).

At CT simulation patients were positioned on a breast board with the ipsilateral arm abducted (Figure 1a). Radio-opaque wire was used to mark the surgical scar and breast and radio-opaque skin markers were set on anterior midline and right or left mid-axilla lines, half-way between the superior and inferior border of the wired breast. The co-ordinates of the LAP laser were recorded to ensure consistent set-up on subsequent CT and US image acquisitions. Standard 3 mm CT slices were obtained using a dedicated CT-Sim (GE Lightspeed, 80 cm aperture). Immediately following acquisition of the planning CT data (CT1), while the patient was instructed not to move, the CT couch was repositioned 500 mm inferior of the CT origin. Scar wires were removed and a 3DUS (US1) was acquired, in ‘Workstation Mode’ , by a radiation therapists using high viscosity gel to minimize breast deformation and optimize image quality.

#### Image guidance (CT2 and US2a, b & c)

Patients returned 14–21 days after their initial planning simulation. All patients were once again placed on the breast board and radio-opaque markers were placed on the skin reference tattoos. Patients were then aligned using the LAP lasers, which were set using the initial planning simulation recorded values. A second CT dataset (CT2) was acquired using the CT simulation protocol described previously. Following CT scanning, the patient was translated 500 mm and three sequential 3DUS image sets (total of 3 US images = US2a, b&c) were acquired by three RTs using the Clarity system in ‘Guide Mode’ (Figure 1b).

### Image registration

CT1 and CT2 image sets were co-registered in ‘Workstation Mode’ by a single radiation oncologist by first applying an automated technique and then by adjusting manually for fine tuning. US2a, 2b, and 2c were automatically co-registered to US1 in Guide Mode using the Clarity in-room coordinate system and LAP laser co-ordinates.

### Seroma contouring

CT1 and CT2 seromas were contoured “off-line” in ‘Workstation Mode’ by a single radiation oncologist experienced in partial breast radiotherapy. Similarly, US1 was contoured “off-line” in ‘Workstation Mode’ by the study RT who performed the initial 3DUS acquisition. US2a, b&c were contoured “off-line” in ‘Guide Mode’ by five RTs involved in the study to reproduce the conditions of 3DUS image guidance at the time of treatment. To promote consistency and speed, RTs were instructed to contour US2, for comparison with US1, using the 5-point semi-automated approach available in ‘Guide-Mode’ using the positioning reference volume from US1 as a guide only.

### Seroma shift calculation

The ‘seroma shift’ represents the change in patient position required to align the seroma at the time of treatment (US2, CT2) to the seroma at the time of CT simulation (US1, CT1). Both CT and US shifts were determined in each of the Cartesian directions (Right/Left, Anterior/Posterior, Superior/Inferior) by comparing the centroid (geometric center of the seroma) contoured at the time of image guidance to the centroid location of the seroma contoured following simulation. Shift calculations were made ‘off-line’. Calculated shifts were not used to position the patient during the actual treatment course.

### Statistical analysis

#### Subjects included in analysis

Subjects were excluded from analysis if they did not meet PBI dosimetric constraints [12] or had poor seroma clarity [11]. Seroma clarity on both CT1 and US1 was assessed using a previously described 6 point scale (0 = no visible seroma to 5 = clear, homogeneous seroma with sharp boundaries) [11]. Each seroma was assigned a consensus score after joint review by a multidisciplinary team (physics, radiation oncology, RT). A seroma clarity of ≤ 2 (seroma identifiable but with significant uncertainties) was deemed too poor for PBI targeting and subjects were excluded from analysis.

#### 3DUS seroma shifts

US seroma shifts were determined by the five RTs on the three subsequent US scans taken at the time of image guidance (Figure 1b). For further investigation a vector defined by a spherical volume of interest with radius = [(R/L)^2^ + (A/P)^2^ + (S/I)^2^]^0.5^ was calculated. This vector defines a three-dimensional region about which the seroma centroid should be located. The vector radius is of clinical interest as it helps define potential target margins.

#### Comparison with CT seroma shifts

Absolute mean shifts in each of the Cartesian directions for seromas contoured on US (US1 – US2) were compared to absolute mean shifts determined by CT (CT1-CT2) for each patient. Shift vectors for seromas contoured on US and CT were compared.

#### 3DUS operator variability

A Two-Way ANOVA at the 95% confidence level (α = 0.05) was applied for each subject. Variability introduced by multiple 3DUS scan acquisitions (scanning variability) was assessed for each patient by comparing the mean centroid displacement between US2a, US2b, and US2c (Figure 1b). Similarly, variability introduced by multiple operators contouring individual US images (operator variability) was assessed by comparing centroid displacement determined between operators on an individual US scan acquisition (Figure 1b). ANOVA was used to determine mean intra-operator and inter-operator seroma shifts for the entire patient cohort and to compare the mean standard deviation (SD) among observers.

#### Time requirements for 3D US IGRT

Estimates were recorded for the time taken to acquire an US image at simulation (US1), to contour the initial US (US1) offline, to acquire subsequent US images (US2a, US2b, US2c), and to contour those images offline.

## Results

### 3D US clarity and PBI eligibility

**Figure 2:**
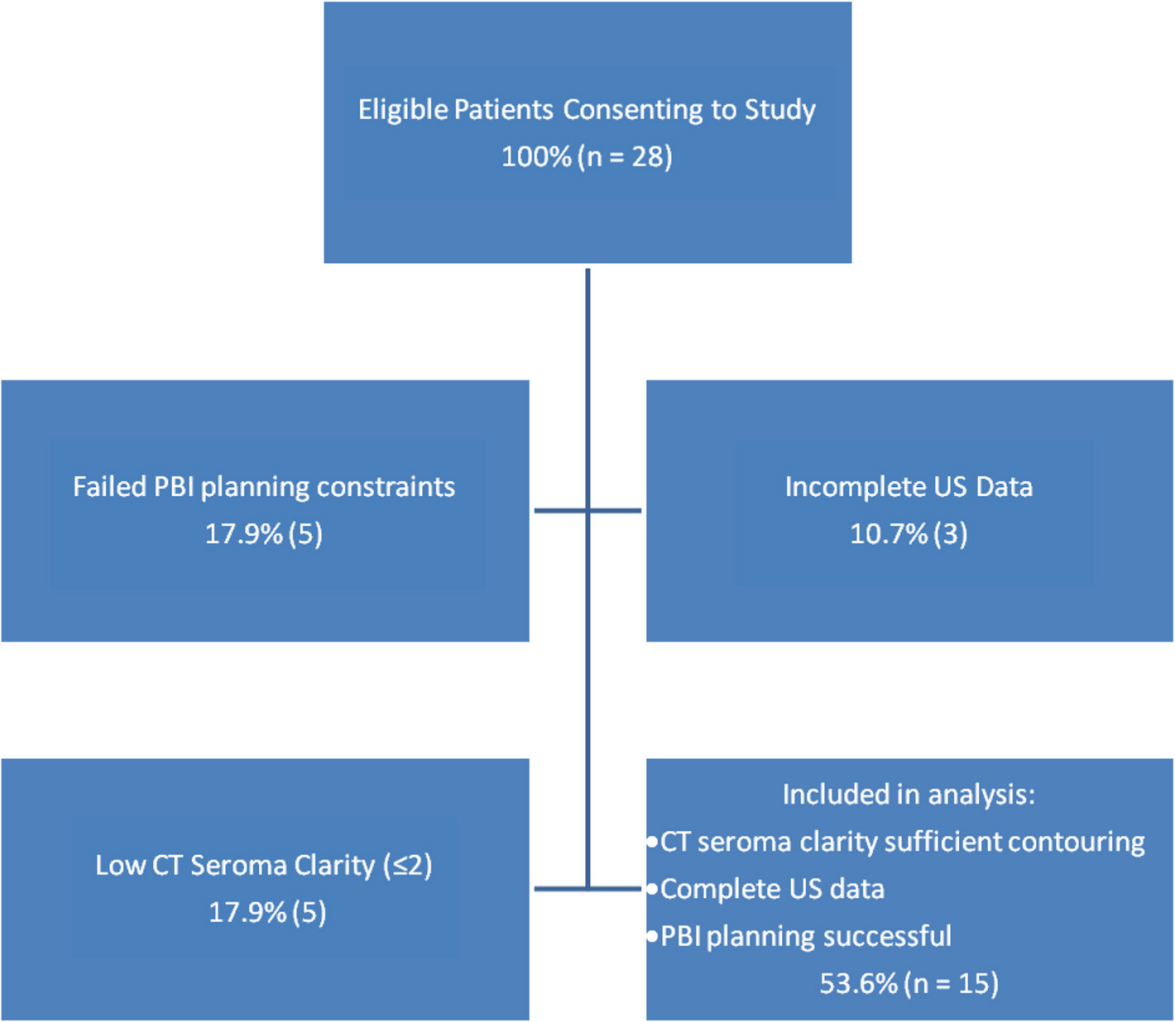
Consort diagram of patients eligible for study, reasons for exclusions and those included in final analysis.

Clinical characteristics and factors affecting PBI planning are outlined in Table 1. Of the 28 subjects who underwent US1/CT1, 13 were excluded due to poor CT seroma clarity, failure to meet dosimetric constraints or incomplete US data acquisition (Figure 2) including one patient with no identifiable seroma at the time of ultrasound, but a CT seroma clarity score of 4. No factors that were predictive of poor CT seroma clarity, PBI planning failure, or poor US seroma clarity, were identified.

**Table 1:** Clinical, pathologic and initial imaging characteristics of consenting subjects (n = 28) and the analyzed subset (n = 15)

	**Entire cohort**	**Analysis cohort**
	**n = 28**	**n = 15**
**Age (years)**		
Median (range)	68 (47–87)	72 (53–87)
**Histology n (%)**		
Invasive Ductal	27 (96)	15 (100)
Ductal Carcinoma in Situ	1 (4)	0
**Pathologic tumour size (cm)**		
Median (range)	1.2 (0.3 – 2.5)	1.4 (0.8 – 2.5)
**Grade (Invasive Ductal) n (%)**		
I	8 (30)	6 (40)
II	16 60)	8 (53)
III	3 (11)	1(7)
**Estrogen receptor status**		
Positive	26 (92.8)	14 (93.3)
**Laterality**		
Right	12 (42.8)	6 (40.0)
Left	16 (57.2)	9 (60.0)
**Seroma location**		
Inner/central	11 (39.3)	5(33.3)
Lower/outer	17 (60.7)	10 (66.7)
**US clarity score**		
Mean	2.7	3.1
**CT clarity score**		
Mean	3.1	3.6
**Breast volume (cc)**		
Mean (range)	1732 (712 – 3877)	1429 (711 – 2088)
**Seroma volume (cc)**		
Mean (range)	32 (7 – 157)	21 (6 – 50)
**Seroma: breast ratio**		
Mean (range)	0.018 ± 0.015	0.0.015 ± 0.013
BCS to CT simulation* (weeks)		
Mean (range)	8.8 (4.4 – 13.4)	9.5 (5.9 – 13.3)

### Magnitude of 3D US shifts

**Figure 3:**
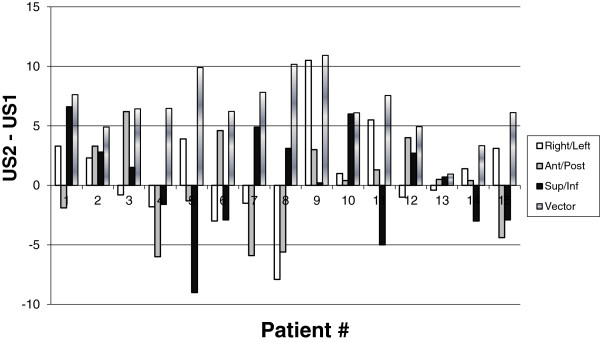
**Mean US seroma shifts and vectors; R/L (white); A/P (grey); S/I (black); Vector (marble) for 15 subjects.** Each shift or vector was the mean of 15 shift calculations per patient (5 RTs × 3US).

Figure 3 shows the mean shifts in each of the Cartesian directions for the 15 subjects using all operator and US data. The minimum and maximum shifts in any direction were 0.2 mm and 10.5 mm respectively. Mean absolute US shifts were 3.1 mm, 3.3 mm, and 3.5 mm for R/L, A/P, and S/I directions respectively. Mean seroma shift vectors ranged from 0.9 mm to 10.9 mm with a mean and standard deviation of 6.6 mm and 2.6 mm respectively. Eight of the 15 patients (53%) demonstrated a shift >5.0 mm in at least one of the Cartesian directions. 10 of the 15 patients (66.6%) demonstrated a vector shift > 5.0 mm and 2 (13.3%) exceeded 10.0 mm.

### Comparison with CT shifts

**Figure 4:**
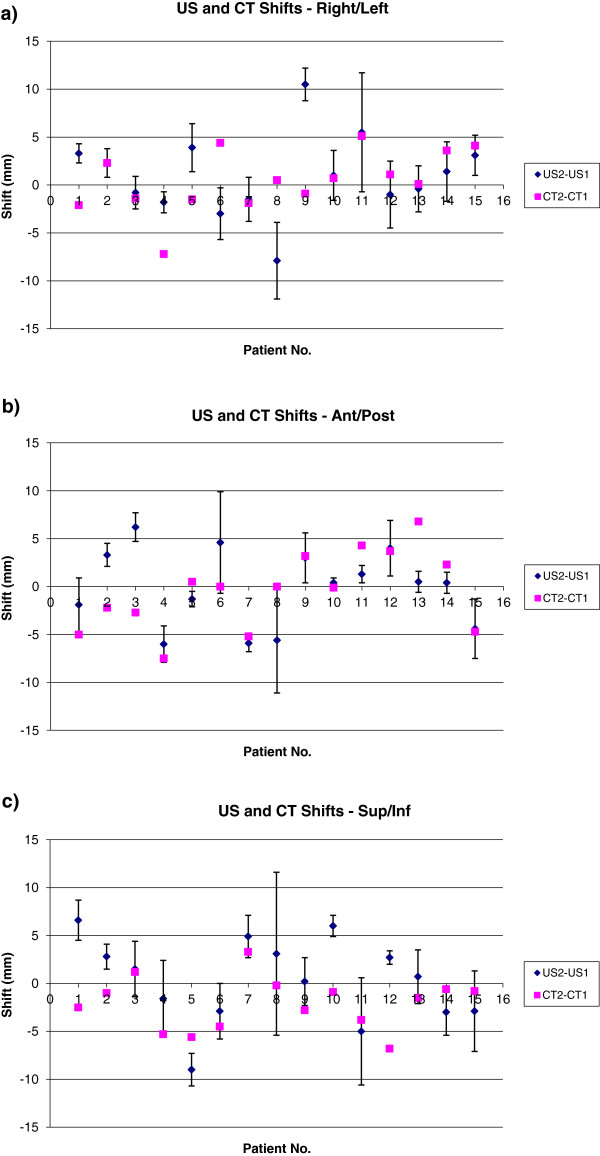
**Comparisons of US and CT shifts in the (a) Right/Left, (b) Ant/Post, and (c) Sup/Inf directions.** Error bars represent the standard deviation among the US2a, 2b, and 2c shifts relative to US1.

Mean shifts for CT1 to CT2 as determined by a single RO were 2.4 mm, 3.3 mm, 2.2 mm for R/L, A/P, and S/I directions respectively and are comparable to shifts as determined by US. Mean seroma shift vectors for CT ranged from 0.5 mm to 11.7 mm with a mean and standard deviation of 5.5 mm and 2.8 mm respectively. Nine subjects (60.0%) demonstrated a vector shift > 5.0 mm and one patient (6.7%) exceeded 10.0 mm when CT was employed for image guidance. The mean and standard deviation for the three Cartesian and the vector shift determined using CT or US were comparable when considering the entire patient population. However, Figure 4 shows that on a patient by patient basis shifts determined using CT or US demonstrated no statistical correlation.

### 3DUS scanning and operator variability

Table 2 shows that seroma shifts determined from multiple scan acquisitions (scanning variability) were statistically indistinguishable (p = 0.42) with an expected standard error in the mean of 0.6 mm in any direction. Over an interval of ± 1.1 mm the seroma shift could be detected with confidence (α = 0.05) on multiple US scans. Seroma shifts determined between operators on the same US scan (operator variability) were statistically indistinguishable (p = 0.19) with an expected standard error in the mean from repeated shift calculations on different US scans being 3.2 mm in any direction. Over an interval of ± 6.2 mm the seroma shift could be detected with confidence (α = 0.05) by different operators on the same US scan.

**Table 2:** ANOVA results for variability in seroma shift determination introduced as a result of multiple US scan acquisitions (scanning variability) and multiple operators contouring on a single US scan (contouring variability)

	**Scanning variability**	**Operator variability**
Standard error in measurement (mm)	0.6	3.2
95% confidence interval (mm)	1.1	6.2
P-value	0.42	0.19

**Figure 5:**
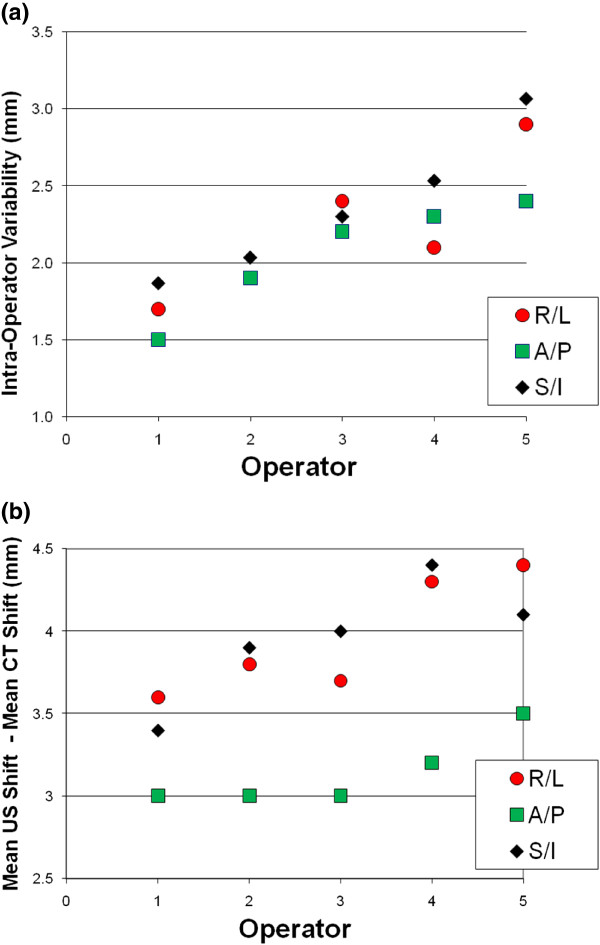
**Comparison of intra-operator variability on US to CT shifts. (a)** An operator effect was observed in the intra-operator variability (shift standard deviation) in seroma shift determination. **(b)** Differences between mean US shifts for each operator and the CT shifts calculated by a single RO demonstrate that the operators with the least variable US results also correlate better with the CT results.

ANOVA was employed to calculate standard deviations in mean seroma shifts. Inter-operator standard deviation means were 2.6 mm, 2.1 mm, and 3.0 mm for R/L, A/P, and S/I directions respectively. Intra-operator SD means were 2.2 mm, 2.1 mm, and 2.8 mm for R/L, A/P, and S/I directions respectively. A substantial operator effect was observed in the intra-operator variability in seroma shift determination (Figure 5a). SD in seroma shifts between operator 1 and operator 5 differed by a factor of almost 2 in all directions. Differences between mean US shifts for each operator and the CT shifts calculated by a single RO demonstrate that the operators with the least variable US results also correlate better with the CT results (Figure 5b).

### Time requirements for 3D US IGRT

The mean and SD time necessary to acquire the US image taken at simulation (US1) was 13 ± 4 minutes. The mean and SD time taken to contour the US1 offline was 7 ± 3 minutes. The mean and SD time taken to acquire and contour each of the US images offline using the semi-automated ‘Guide Mode’ as the surrogate for treatment (US2a, US2b, US2c) was 11 ± 4 minutes.

## Discussion

One concern for the use of US as a tool for image guidance is that US scanning involves a certain pressure that can cause the breast tissue to deform at the time of imaging. However, several studies have examined the efficacy of 3DUS for daily localization of the surgical bed and have reported good correlation of 3DUS with CT in its ability to localize and track target displacements [10,11]. The current study sought to determine the extent to which operator variability in 3DUS acquisition and seroma contouring contributed to seroma shift differences calculated for breast IGRT.

In clinical practice it is likely that multiple operators would be involved with the acquisition, contouring, and patient repositioning if 3DUS IGRT was utilized. There are many factors that may confound the accuracy of the localization of the target between fractions. We have defined “scanning” variability to encompass those potential sources of variability related specifically to image acquisition. Scanning variation included differences in US scan settings, patient breathing during image acquisition, probe pressure [12] and scanning direction. Scanning variability was assessed by comparing seroma shifts between multiple 3DUS scans. Similarly, we have defined “operator” variability to encompass those potential sources of variability related specifically to the seroma contouring. Contouring variation included image window and level settings, contouring strategy, and selection of the interpolation algorithm used for 3D contour extrapolation. To reduce contouring variation, the 5-point semi-automated approach, which proved to be the most consistent and accurate contouring method in a preliminary study, was chosen for use in this study. Despite this attempt to standardize the approach to contouring our results show that contouring variability contributes more significantly to the overall variability in seroma localization than the variability associated with multiple scan acquisitions. The data suggest that at the level of 95% confidence contouring variability contributes a mean uncertainly of 6.2 mm in the determination of the seroma location compared to a mean uncertainty of 1.1 mm contributed by the variability from multiple scan acquisitions.

Our results demonstrate a clear difference between the RT operators that participated in the study. Operators with the least variable 3DUS results had better correlation with seroma shifts determined using CT. Operators all had similar levels of experience, were all trained in the same manner, and were not systematically monitored throughout the scan acquisition or contouring. Therefore, suggesting what technique or process allowed some therapists to systematically arrive at better results than others would purely be speculative. However, the factor of two observed in the standard deviation in seroma shifts between the most extreme operators provides sufficient motivation to further explore inter-operator procedural differences to improve the consistency of 3DUS IGRT in practice.

In our study, the mean and SD seroma shift vectors were 6.6 ± 2.6 mm and 5.5 ± 2.8 mm for 3DUS and CT respectively. While this is suggestive of good correlation between 3DUS and CT it disguises substantial variation between the 3DUS and CT shifts of an individual patient. In fact, there was no correlation observed between 3DUS and CT when comparing individual Cartesian shifts. The seroma shifts determined using 3DUS, however, are comparable to CT being mindful that any discrepancies observed are more reflective of measurement “noise” than true differences between imaging modalities.

The mean seroma shift should inform an adequate PTV margin necessary to ensure proper coverage when IGRT is not used. Wong *et al.*[10] used 3DUS IGRT for 20 patients and measured an average target displacement of 10.8 ± 6.3 mm from the treatment plan. The authors suggest that a margin of 23.4 mm (mean plus 2 SD) may be needed for electron boost or PBI to ensure the target is covered ≥ 95% of the time when IGRT is not used. We measured a mean seroma shift vectors of 6.6 ± 2.6 mm. This suggests that a margin of 11.8 mm may be needed for electron boost or PBI to ensure that the target is covered ≥ 95% of the time when IGRT is not used. It should be recognized, however, that of the 15 patients in our study 10 (66%) demonstrated a vector shift > 5.0 mm and 2 (13.3%) exceeded 10.0 mm. Suggesting that a much larger margin is required in the absence of IGRT. Additionally, while the margins suggested by our results improve on the results of Wong et al. [10] by almost a factor of two, this may be attributed to stricter patient eligibility in our study.

The seroma shifts observed in our study were in many cases in excess of the CTV to PTV growth used by Kirby *et al.*[7] and those used in ongoing trials evaluating external beam [13]. This emphasizes the need for daily image guidance for PBI to avoid unreasonably large margins or under-treatment of the CTV.

## Conclusions

Variability introduced by multiple scan acquisitions or by multiple operators contouring 3DUS images does not contribute significantly to seroma shifts. Seroma shifts for IGRT determined using 3DUS are of similar magnitude to CT. 3DUS IGRT requires approximately 15 minutes at simulation and 11 minutes per IGRT fraction at treatment. An observed inter-operator effect suggests future work to minimize variability and may improve the efficacy of 3DUS as an IGRT tool for breast PBI.

## Competing interests

This research was funded by a grant to Dr. Tanya Berrang from *Clarity*^*TM*^, Elekta Soft Tissue Visualization, Montreal, Quebec. ClarityTM did not have any role in the analysis or reporting of the results.

## Authors’ contributions

AL, TB, and IG contributed to study design, data analysis, and preparation of the final manuscript. TB, TM, HV, LS, SS, and JW carried out data acquisition. CP, IO, and WB provided technical support with the study design and the interpretation of results. All authors read and approved the final manuscript.
